# Work and Wellbeing in the 21st Century †

**DOI:** 10.3390/ijerph13111065

**Published:** 2016-10-31

**Authors:** Paul Litchfield, Cary Cooper, Christine Hancock, Patrick Watt

**Affiliations:** 1 BT Group plc, BT Centre, London EC1A 7AJ, UK; paul.litchfield@bt.com; 2 Manchester Business School, University of Manchester, Booth Street West, Manchester M15 6PB, UK; 3 C3 Collaborating for Health, London SE1 4YR, UK; christine.hancock@c3health.org; 4 Bupa UK, London WC1A 2BA, UK; patrick.watt@BUPA.com

## 1. Introduction

The nature of work and the way it is conceptualised has been evolving since the dawn of humankind. As societies have shifted from hunter gathering to an agrarian basis and then to urban living, the activities that people have undertaken have changed. In parallel, the arrangements for the delegation of labour have altered from systems such as slavery, serfdom and indentured labour to paid employment and contracting. The pace of change has been increasing exponentially and the information technology revolution has transformed work for many in less than a generation.

Attitudes and behaviours tend to adjust more slowly than technological advances. The societal view of work in more developed countries remains largely based on the experiences of the 19th and 20th centuries. The label “the Industrialised World”, still often used to describe more affluent nations, is itself a reflection of a failure to come to terms with the reality that we live in a post-industrial society. The most physically hazardous work has largely been rendered redundant, engineered out or (shamefully) exported to less developed parts of the world. The knowledge economy has created millions of jobs in sectors which did not exist 30 years ago but the way work is organised and the management practices employed often reflect a bygone age. It is over 100 years since the Liberal Reforms, driven through by Churchill and others, introduced compensation for industrial injury. That is a term still enshrined in the UK’s social security system, conjuring up a picture of bodies broken by heavy machinery, but the greatest harm caused by work in modern society is psychological—minds shattered by brutish systems designed and applied carelessly.

Some organisations have responded to these changing trends and have sought to mitigate the potential harm to mental as well as physical health from bad work. Rather fewer have sought to promote good health as a way of engaging the workforce and promoting higher productivity. Only a tiny minority have embraced the notion of wellbeing as an enabler for sustained commercial success and as a measure of their wider impact on society [1].

## 2. Wellbeing

The term “wellbeing” is imperfect. It has the advantage of being understood intuitively (at least in the English speaking world) as referring to “how we are doing” as individuals, communities or society but its broad coverage renders simple definition problematical. This is compounded by the different slants taken by those from an economic, health and social science background—all of whom have an interest in the subject—and the trivialisation of the issue in some parts of the media. A number of organisations, including some companies like AstraZeneca, BT, Shell, Unilever, Nestlé, have developed definitions with supporting measures in recent years. The UK Office for National Statistics has produced, arguably, the most comprehensive model [2]. This encompasses 10 domains and 43 measures but the domain most relevant to the workplace is personal (or subjective) wellbeing. Personal wellbeing can be thought of as life satisfaction based on an individual’s perception of their health, happiness and sense of purpose [3]. It is this narrower element of personal wellbeing, and its interaction with work, which will be considered throughout the remainder of this piece.

## 3. The Evolution of Knowledge

Work has long been recognised as having both positive and negative influences on health and wellbeing. In ancient Greece, Galen wrote that employment is “nature’s physician, essential to human happiness” while his near contemporary the Roman scholar Pliny first described mercury poisoning among slaves working in the mines. The Industrial Revolution brought many benefits to society but it also introduced new ailments which, because they were concentrated in specific occupational groups, could be recognised by the emerging science of medicine. So it was that scrotal cancer was identified in chimney sweeps, byssinosis in cotton workers and “phossy jaw” in matchmakers. There was a growing realisation during the 19th and 20th centuries that various chemical, physical and biological agents encountered in the workplace could cause harm to the health of those exposed. The response was to introduce a raft of legislation specific to hazards and the industries in which they were encountered. By the second half of the 20th century it became clear that this approach was cumbersome, overly reactive and inconsistent. A more comprehensive framework was therefore introduced based on a risk management hierarchy of eliminating hazards where possible and substituting them with less harmful alternatives or controlling exposures where use was essential. This strategy has proved effective and many industrial diseases are no longer seen or are a legacy of employment many decades ago.

There continue to be hazards in the modern workplace but they relate more often to the way that work is organised rather than specific agents and the consequential harm is more psychological than physical. The UK Health and Safety Executive has taken a lead in commissioning research to understand the work factors that can affect psychological health and in the early part of the 21st century published management standards for organisations. The standards cover the primary sources of stress at work which are:

- Demands—this includes issues such as workload, work patterns and the work environment.
- Control—how much say that the person has in the way they do their work.
- Support—this includes the encouragement, sponsorship and resources provided by the organisation, line management and colleagues.
- Relationships—this includes promoting positive working to avoid conflict and dealing with unacceptable behaviour.
- Role—whether people understand their role within the organisation and whether the organisation ensures that they do not have conflicting roles.
- Change—how organisational change (large or small) is managed and communicated in the organisation.

The standards are helpful but highlight the deficiencies of the established risk management hierarchy in the area of psychological health. That traditional model works well for agents that are only ever hazardous to health but falls short when activities may be beneficial or harmful in different contexts. It is similarly limited in dealing with health issues, physical or psychological, that may not be caused by work but which impact on capability and therefore manifest in the workplace to the detriment of the individual and the employer.

## 4. The Harm/Benefit Paradox

It is clear that work can be harmful but so can the absence of work. The link between poverty and illness has been recognised for many centuries but it was only in the 1930s that the independent effect on health of unemployment was first described [4]. Research since that time has confirmed that both job loss and continuing ”worklessness” impact adversely on people’s health with increased levels of both mental and physical problems. Rates of anxiety, depression [5], suicide [6], hypertension [7], diabetes, stroke and heart attack [8] have all been shown to be elevated in those who are made unemployed. There is therefore a balance to be struck between the good and bad aspects of work and the seminal evidence review was carried out by Wadell and Burton in 2006 [9]. The authors found that overall the beneficial effects of work outweigh the risks and are greater than the harmful effects of long-term “worklessness”. They therefore concluded that work is generally good for health and well-being. This statement has since been refined to advocate the concept of “good work” in which the harmful aspects are avoided but the psychosocial benefits of purpose and social identity are retained.

## 5. The Changing Pattern of Health

The medical advances of the 20th century have transformed the global disease burden. Infectious diseases remain an ever present threat but for the time being morbidity and mortality are dominated by non-communicable disease. Heart disease, cancer, diabetes and chronic respiratory disease have become the leading cause of death world-wide. More importantly from an employment perspective, the rising prevalence and reducing age of onset mean that incapacity and mortality are increasingly affecting the working age population. In parallel, mental health disorders have become the most important cause of disability in all WHO regions, accounting for around one third of Years Lost to Disability (YLD) among adults aged 15 years and over [10]. Furthermore, unlike most chronic illnesses, the age distribution is relatively constant with adults of working age being as likely to suffer as those who are older. The economic cost to society is substantial with depression alone estimated as absorbing 1% of Europe’s GDP [11]. For individual companies mental health is now often the commonest cause of sickness absence in developed countries, accounting for up to 40% of time lost [12] with presenteeism adding at least 1.5 times to the cost of absenteeism [13].

Much of this ill health is driven by lifestyle changes and interventions that can turn the situation around are generally low key and inexpensive. Work can be a contributory factor and the role of the employer in addressing excess sedentary employment or psychological pressures is obvious. However, the workplace is also an effective venue for more general health promotion, especially when targeting hard to reach groups like men, and group interventions tend to have better outcomes than those delivered individually.

## 6. The Changing Pattern of Work

Work is changing, both in the nature of tasks undertaken and in the way that activities are organised. The global drift of populations to cities, the increasing proportion of women in the workforce and the emergence of a 24/7 culture has disrupted traditional patterns of work life balance and social support mechanisms. The changing roles of men and women at work has had dramatic impact on how people are managed, the right to request flexible working, the long hours culture, the glass ceiling for women and other diversity issues in the workplace. The technological revolution has transformed not only what many people do for a living but also the way that they work. Technology can be used and viewed as a liberating force enabling workers to juggle increasingly complex demands or as an oppressive influence that removes discretion and denies the worker any respite from his labours. Recent research is suggesting that emails and other social media may actually be electronically overloading people as well as interfering with their non-work lives, adversely affecting not only the health of workers and their families but also undermining productive work. The ways that work is organised and the uses made of technology are therefore critical to the wellbeing of both individuals and societies.

## 7. A Case for Action

There are clear benefits to individual workers from promoting wellbeing in the workplace, both in terms of the quality of the work itself and the provision of an environment that encourages healthy behaviours. Society is also a beneficiary in terms of increasing the stock of healthy, motivated citizens who are available to support their communities and who are less likely to be a drain on State resources. However, the case for employers to invest in this area has been less well evidenced—even a healthy return on investment is less attractive if the benefits do not accrue to those who incur the costs. In countries such as the USA, where the employer generally funds healthcare, the financial case is more straightforward and much of the evidence for “wellness” programmes comes from there. The emphasis has largely been on preventative measures to reduce the disease burden, and hence the treatment costs, of chronic physical diseases rather than mental health problems that tend to impact less on health insurance premiums.

In countries with predominantly State healthcare provision, like the UK, a reduction in use of services produces no direct benefit to most employers and the focus has been more on interventions to limit other costs such as sickness absence. Inevitably, that creates a bias towards conditions that result in extended or repeated absence rather than those that necessarily cost a lot to treat. Mental health has therefore been more prominent as a workplace issue in the UK than it has in the USA. The business case for intervention has largely been founded on cost control with the Confederation of British Industry (CBI) quoting an average direct cost for each absent employee of £975 per annum [14]. Indirect costs such as management time, lost output, reduced service quality and replacement costs are cited but less often quantified.

There has been a growing awareness that the business benefits from improving wellbeing can be much greater than just cost control. The productivity gap between the UK and other advanced economies is significant and increasing [15]. There are many factors that influence productivity but worker wellbeing can certainly impact on economic output [16]. There is limited evidence from the business world but some published work does indicate that interventions can have a material and significant effect on commercial outcomes [17]. Also important in this context is the indirect benefit of wellbeing on the recruitment and retention of key staff, something known in HR parlance as “regrettable (labour) turnover”.

Taking an interest in employee health is a potent driver of workforce trust and improving levels of wellbeing has been shown to be associated with more sustained levels of engagement and performance [18]. There is some debate about the causal direction of this association but the literature suggests three mechanisms by which higher levels of wellbeing can drive higher performance [19].

- Impacting on cognitive abilities and processes—enabling workers to think more creatively and be more effective at problem solving
- Influencing attitudes to work—increasing workers’ propensity to be co-operative and collaborative
- Improving physiology and general health—giving workers greater levels of stamina and resistance to illness

These mechanisms assume an even greater importance as we move further and faster into a knowledge based economy.

## 8. A New Framework for Workplace Wellbeing

**Figure 1:**
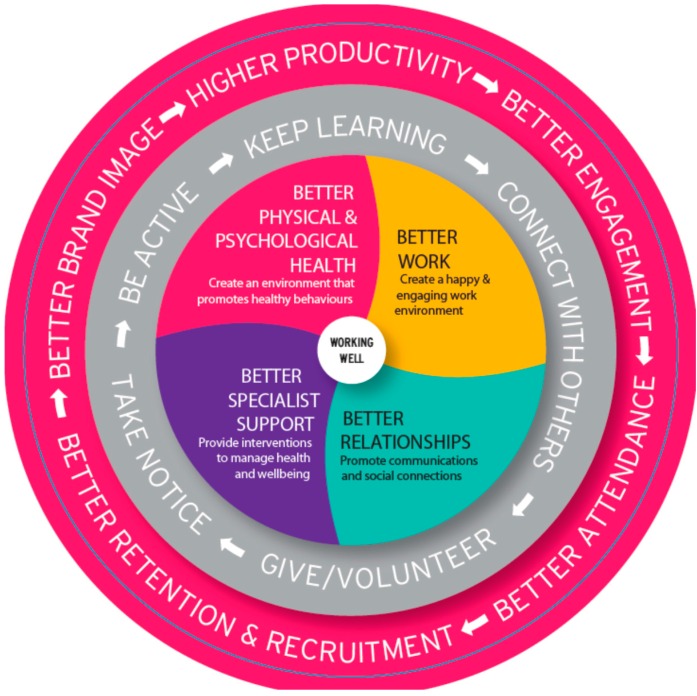
Business in the community “Workwell Model”.

Thinking has evolved to take account of these changes and many believe that what is required is an integrated approach which puts wellbeing at the heart of a company’s people agenda. The evidence indicates that benefits accrue from this approach by taking action at both an organisational and at an individual employee level. The UK organisation Business in the Community has created the “Workwell Model” [20] to demonstrate the business benefits for employers who take a proactive approach to the prevention of illness, the promotion of wellbeing and a focus on the quality of work. The model (Figure 1) also promotes early intervention for employees who develop a health condition and active sickness absence management to rehabilitate people back into work.

The framework has been adopted by a number of companies and public reporting against it has been well received by institutional investors. Signatories to the Principles of Responsible Investment initiative [21] manage some $59 trillion of assets and cite application of this sort of human capital management as an indicator of longer term financial performance.

## 9. Work Design

Work design is critical in determining the psychological reaction people have towards what they are doing and, hence, the satisfaction they derive, their motivation and their performance. One influential framework for addressing this issue is the Job Characteristics Model developed by Hackman and Oldham [22] which identifies five core job characteristics:

- Skill variety. Making use of an appropriate variety of skills and talents for a given individual worker—neither too many as to be overwhelming nor too few leading to boredom.
- Task identity. Being able to identify a recognisable outcome from the task undertaken, either as an individual or part of a group, so that the worker can feel a sense of achievement and pride in what has been done.
- Task significance. Seeing that the task has a beneficial impact on others, over and above the worker himself or herself, either within or outside the organisation.
- Autonomy. Allowing the worker to exercise a degree of freedom, independence and discretion in the way work is scheduled and the process by which it is carried out.
- Feedback. Providing information on how effective the worker has been in converting effort into performance (so that mistakes can be learned from) and connecting the worker emotionally with the end user of his output.

This approach has been used successfully in many countries to improve organisational performance, quality and profitability through paying attention to the feelings, motivation and job satisfaction of workers. Less sophisticated approaches that focus solely on outcome measures such as production levels or profit margins, while neglecting the wellbeing of the people employed, tend to produce unhappy workplaces where individuals burn out and success cannot be sustained.

## 10. Management Competencies

The way that people are managed at work has a profound influence on their wellbeing. It is a sad reflection of some modern management practice that many workers report the worst time of day as being when they were with their line manager [23]. Conversely, it is a consistent finding of employee engagement surveys that the first line manager is the most trusted source of information in the workplace. The reality is that there are good and bad managers but, commonly, people are not selected for management roles on the basis of their people skills and they are often given little or no training in how to handle interpersonal relationships. Some companies are seeking to redress this shortcoming and a set of management competencies has been developed by the UK Health and Safety Executive [24], in conjunction with the Chartered Institute of Personnel and Development and Investors in People, to define the behaviours identified as effective for preventing and reducing stress at work—see table (Table 1).

Understanding and good communication are central to the management competencies and demographic changes in the workplace are producing new issues to consider. The growing number of working women, the unprecedented levels of migration into the UK and the extension of working lives leading to potentially 4 generations being represented in the workforce create cultural challenges as well as practical issues for those managing organisations.

## 11. Agile Working

Technology can be an enabler for different ways of working but it is mind sets that need to be updated if work is to be organised so as to realise the benefits to wellbeing while meeting business needs. The term “flexible working” has become tainted in some circles and can be interpreted as solely an employee benefit, applied inflexibly and perceived as an unwelcome cost to business. The Agile Future Forum, an initiative by some of the largest businesses in the UK, has helped to reframe “agile working” practices that have mutual benefit and are therefore sustainable. The practices are defined along four dimensions [25].

- Time: when do they work? (e.g., part time, shifts, staged retirement)
- Location: where do they work? (e.g., homeworking, across multiple sites)
- Role: what do they do? (e.g., multi-skilling, secondments, job rotation)
- Source: who is employed? (e.g., permanent employees, outsourcing, crowd sourcing)

Guidance from the Chartered Institute of Personnel & Development (CIPD) [26] highlights the improvements to work life balance and job satisfaction that can result from agile working. Agile teams are characterised by self-organisation, iteration, customer centricity, knowledge sharing, collaboration and mutual trust. These characteristics embody both elements of the Job Characteristics Model and the Five Ways to Wellbeing [27].

The balance between individual and organisational needs must be maintained if wellbeing is not to suffer. There is a powerful link between job insecurity and low well being [28] and some developments in agile employment practice (such as serial short term appointments or zero hours contracts) can carry risks in this regard. Practices such as set working hours and a job for life may be inappropriate for modern society but most people need some certainty in their employment and income. Some societies have attempted to simultaneously strengthen flexibility and security for the benefit of both parties in an employment relationship, by finding a balance between the rights and responsibilities of employers, workers and the authorities. This concept of “flexicurity” embodies four components:

- Flexible and reliable contractual arrangements
- Comprehensive lifelong learning strategies
- Effective active labour market policies
- Modern social security systems

Surveys of citizens’ perception of their own well-being consistently show that levels are highest in countries, such as those of northern Europe, where flexicurity is an accepted part of the socio-economic system [29] and the principles have been adopted by the European Union as part of its Employment Strategy.

## 12. Managing Change

One consequence of the rapid advance of technology coupled with the demands of a market driven by short term interests is that the pace of change in organisations has accelerated in this century. Mergers and acquisitions disrupt organisational structures and culture and, even in “steady state”, reorganisations are a regular feature in almost every large company. Major restructuring, particularly when associated with insecurity, affects the psychological contract between the organisation and its employees and the trust on which that contract is based [30]. This, in turn, results in lower levels of organisational commitment by the workforce especially when associated with feelings of inequity about the restructuring process [31]. Unsurprisingly, job satisfaction levels tend to fall and social relationships among colleagues and with superiors deteriorate [32]; this may manifest as higher levels of bullying and harassment. The evidence relating to performance is more equivocal though the impact of restructuring is generally negative; in a minority of studies productivity was found to rise but, even here, the quality of work was lower [33]. Similar effects are seen in occupational safety where accident rates tend to rise with job insecurity as workers are inclined to become less compliant and adopt more risk taking behaviours [34].

Restructuring is a change issue and application of the fundamental principles of effective leadership and sound change management will reduce harm to individuals and the organisation. However additional measures have been shown to be beneficial and may form the basis of a future European Framework agreement. A key theme running through the evidence is the requirement for *Perceived Justice* (i.e., not just that the organisation does act fairly but that those affected by the restructuring believe that to be the case).

There are three key dimensions that influence whether individuals accept that change is being implemented fairly [35].

Distributive justice. The selection criteria for the individuals and parts of the organisation subject to changes. This incorporates elements related to “needs”, such as legislation and collective agreements, as well as “efficiency” which captures skills, productivity, etc.

Procedural justice. This requires consistent procedures executed without bias and on the basis of accurate information. There must be a trusted mechanism for correcting poor decisions and the system must be founded on ethical and moral standards appropriate for the society in which the organisation is operating.

Interactional justice. The nature and timing of internal and external communication is vital in maintaining trust. Clear, early, open and personal communication with those involved is critical in avoiding uncertainty, rumour and de-motivation.

Paying attention to these elements and ensuring that, in particular, middle managers are engaged increases the likelihood of successful change management. Middle managers are the element in an organisation that “buffers chaos” translating strategic intent into practical action. They need to be persuaded of the case for change at an emotional as well as an intellectual level so that they can take shared ownership of the issues.

## 13. The Ageing Workforce

In almost all countries of the world people are living longer on average and are remaining healthy and capable to a later age. Most of today’s 65-year-olds will live beyond 80, and some will live beyond 110 [1] placing a significant extra load on pension schemes and the social security system. The cost of dementia alone to the UK in terms of treatment and lost productive value would approximate to £50b per annum. This is happening at a time of generally falling birth rates, shifting the age demographic from its traditional pyramid to a more cylindrical shape. The demands for a more educated population have deferred the entry age into work further compounding labour shortages. Current UK employer plans suggest that we need to fill 13.5 million job vacancies in the next ten years, but only 7 million young people will leave school and college. These factors have resulted in a mature and ageing workforce in most sectors. Those aged over 50 already form 27% of the UK workforce and by 2020 it will be a third, while more than 1m people aged over 65 are still working. The rising state pension age, the destruction of defined benefit pension schemes and increased care commitments at both ends of life make it inevitable that the trend to “working later” will continue.

This creates both opportunities and problems in the workplace. Will people working into their 70s create blockages for younger staff? Will we have to change the nature of the job that older workers do to enable them to work in challenging physical and emotional jobs? Will career paths have to change to accommodate the aging workforce? Are there certain types of jobs that would be more suitable for older workers? Can we use experienced older workers to act as mentors to the young workers? How will the intergenerational workforce get on with their markedly different perceptions, aspirations and means of communicating? These are all questions that society needs to tackle and with some urgency [36].

## 14. Conclusions

Wellbeing has moved centre stage in recent years as those concerned with the development of a meaningful and sustainable society have become increasingly dissatisfied with purely financial measures of progress. That shift has been mirrored in the workplace where business leaders are seeking a purpose for their organisations over and above maximising return on capital and realising that achieving such a position in itself helps to deliver commercial success. Human capital management is now seen by many, including the investor community, as an indicator of companies’ long term prospects—the wellbeing of an organisation and the wellbeing of its workers are inextricably linked. There is a growing body of evidence to show which activities influence wellbeing at work, for better and worse, but many gaps remain. As the 21st century progresses, it seems likely that workplace wellbeing will continue to climb up the business agenda and, as it does so, employers will play an increasingly important role in determining the state of the human condition. The reforms that Churchill and his colleagues introduced at the beginning of the 20th century are a platform on which his successors can build.

This Special Issue on occupational stress, human health and wellbeing is building on the early work of Winston Churchill in creating enhanced wellbeing in the workplace. We have published 16 papers from distinguished scholars across the globe. These papers cover many of the workplace stressors such as work engagement, bullying at work, work-family conflict, impaired work ability through musculoskeletal issues and co-worker/managerial sources of stress. We also explored the stresses and strains associated with police work, teaching, medicine and engineering/technical work. And finally we had papers which provided data on the efficacy of coping interventions, stress prevention, resolving work-family conflict, enhancing psychological capital and improving the quality of working life. Remember the words of Studs Terkel in his acclaimed book *WORKING*, “Work is about a search for daily meaning as well as daily break, for recognition as well as cash, for astonishment rather than torpor, in short, for a sort of life rather than a Monday through Friday sort of dying”.

## Figures and Tables

**Table 1:** Behaviors that prevent or reduce stress at work.

Competency	Sub-Competency
Respectful and responsible: Managing emotions and having integrity	Integrity *Being respectful and honest to employees*
	Managing emotions *Behaving consistently and calmly around the team*
	Considerate approach *Being thoughtful in managing others and delegating*
Managing and communicating existing and future work	Proactive work management *Monitoring and reviewing existing work, allowing future prioritisation and planning*
	Problem solving *Dealing with problems promptly, rationally and responsibly*
	Participative/empowering *Listening to, meeting and consulting with the team, providing direction, autonomy and development opportunities to individuals*
Managing the individual within the team	Personally accessible *Available to talk to personally*
	Sociable *Relaxed approach, such as socialising and using humour*
	Empathetic engagement *Seeking to understand each individual in the team in terms of their health and satisfaction, motivation, point of view and life outside work*
Reasoning/Managing difficult situations	Managing conflict *Dealing with conflicts decisively, promptly and objectively*
	Use of organisational resources *Seeking advice when necessary from managers, HR and Occupational Health*
	Taking responsibility for resolving issues *Having a supportive and responsible approach to issues and incidents in the team*
